# Understanding Fatal and Non-Fatal Drug Overdose Risk Factors: Overdose Risk Questionnaire Pilot Study—Validation

**DOI:** 10.3389/fphar.2021.693673

**Published:** 2021-09-28

**Authors:** Radhouene Doggui, Keyrellous Adib, Alex Baldacchino

**Affiliations:** ^1^ Chronic Disease Prevention Research Laboratory, Centre de Formation Médicale du Nouveau Brunswick, Université de Sherbrooke, Sherbrooke, QC, Canada; ^2^ Faculty of Medicine and Health Sciences, University of Nottingham, Nottingham, United Kingdom; ^3^ Division of Population and Behavioral Science, University of St Andrews School of Medicine, Scotland, United Kingdom

**Keywords:** fatal overdose, exploratory factor analysis, risk factors, adults (MeSH), Scotland

## Abstract

**Background:** Drug overdoses (fatal and non-fatal) are among the leading causes of death in population with substance use disorders. The aim of the current study was to identify risk factors for fatal and non-fatal drug overdose for predominantly opioid-dependent treatment–seeking population.

**Methods:** Data were collected from 640 adult patients using a self-reported 25-item Overdose Risk (OdRi) questionnaire pertaining to drug use and identified related domains. The exploratory factor analysis (EFA) was primarily used to improve the interpretability of this questionnaire. Two sets of EFA were conducted; in the first set of analysis, all items were included, while in the second set, items related to the experience of overdose were removed. Logistic regression was used for the assessment of latent factors’ association with both fatal and non-fatal overdoses.

**Results:** EFA suggested a three-factor solution accounting for 75 and 97% of the variance for items treated in the first and second sets of analysis, respectively. Factor 1 was common for both sets of EFA analysis, containing six items (Cronbach’s *α* = 0.70) focusing around “illicit drug use and lack of treatment.” In the first set of analysis, Factors 2 (Cronbach’s *α* = 0.60) and 3 (Cronbach’s *α* = 0.34) were focusing around “mental health and emotional trauma” and “chronic drug use and frequent overdose” domains, respectively. The increase of Factor 2 was found to be a risk factor for fatal drug overdose (adjusted coefficient = 1.94, *p* = 0.038). In the second set of analysis, Factors 2 (Cronbach’s *α* = 0.65) and 3 (Cronbach’s *α* = 0.59) as well as Factor 1 were found to be risk factors for non-fatal drug overdose ever occurring. Only Factors 1 and 3 were positively associated with non-fatal overdose (one in a past year).

**Conclusion:** The OdRi tool developed here could be helpful for clinical studies for the overdose risk assessment. However, integrating validated tools for mental health can probably help refining the accuracy of latent variables and the questionnaire’s consistency. Mental health and life stress appear as important predictors of both fatal and non-fatal overdoses.

## Introduction

The rates of drug-related deaths (DRDs) and non-fatal drug-related overdoses (ODs) of opioid users are increasing (Iversen et al., 2016). Illicit and licit drug overdose is a leading cause of premature death and morbidity among this population (Darke et al., 2003; Iversen et al., 2016). Worldwide, overdose-related mortality accounts for 0.65 (0.55–0.75) per 100 person-years, followed by trauma and suicide-related deaths, with values of 0.25 and 0.12, respectively (Degenhardt et al., 2011). In Scotland, 49% of the drug treatment seeking population had experienced a drug overdose at some time in the past and 11% had overdosed in the past 3 months (Bohnert et al., 2011).

A review of the risks of fatal drug overdose in opioid users identified the following three key components (Frisher et al., 2012): 1) individual—relating to the drug (licit or illicit) users; 2) situational—circumstances surrounding an overdose; and 3) organizational—the response to an overdose incidence.

Taken together, these components lead to a complex set of risk factors which will influence the likelihood of a drug overdose occurrence being fatal (European Monitoring Centre for Drugs and Drug Addiction, 2015). Given the premise that multiple variables will influence the risk of drug overdose, it is important to develop preventative measures which can take account of multiple components and provide a more tailored approach to opiate overdose. To date, research has focused on identifying individual person-centered characteristics and circumstances as risk factors. The severity of dependence, recent prison release, recent detoxification, polysubstance use, social deprivation, history of suicide attempt, recent hospital discharge, length of drug using career, number of network members who inject drugs, lifetime history of negative life events, male gender, and homelessness have all been reported as risk factors for fatal opioid-related overdoses (Wolff, 2002; Neale and Robertson, 2005; Coffin et al., 2007; Rome et al., 2008; Backmund et al., 2009; Bohnert et al., 2010; Merrall et al., 2010; Jenkins et al., 2011; Frisher et al., 2012; Mathers et al., 2013).

However, the relative impact of these factors on overdose risks, or how the factors may combine to predict the risk of experiencing a fatal drug overdose, remains poorly determined. Despite the considerable scope of the problem, the independent predictive factors for opioid-related drug overdoses have not been the subject of robust methodological evaluation (Laupacis et al., 1997; McGinn et al., 2000; Reilly and Evans, 2006). This problem is likely to get worse given the aging population of opioid drug users in the United Kingdom (Public Health England, 2016). A recent survey of 123 drug users over 35 years found 75% had overdosed at some point in their lives and 33% in the last 12 months. Extrapolation to the drug using population in Scotland estimated that 4,500 drug users aged over 35 years will experience an overdose event annually (Matheson et al., 2019). As this group has multiple health challenges and problems of social isolation, the number of fatal overdoses should be expected to increase.

Perception of risk is conceptualized in terms of 1) personal vulnerability to the health effects of their risky behavior through knowledge acquisition (Kotchick et al., 2001), 2) “optimistic bias” (inaccurate estimation of lower personal risk in comparison to other counterparts), and 3) “precaution effectiveness” (believing that engaging in precautionary behavior will be beneficial to their health) (Peretti-Watel, 2003). As a result, this cognitive process could increase vulnerability to drug overdose.

For overdose prevention and response research, a broad assessment capable of capturing behavioral risks in populations with varying substance choices and use patterns is critically important, particularly as we seek to understand the precipitants of changes in overdose risk behaviors among at-risk populations. To better understand the factors that cause opioid-related overdose, a first step is to comprehensively assess overdose risk behaviors and test their associations with overdose events.

One difficulty in preventing fatal as well as non-fatal drug overdoses is that the risk factors for such episodes are not well understood, and therefore, at-risk individuals cannot be reliably identified and interventions cannot be targeted at those most at risk. To date, research has focused on identifying isolated characteristics and circumstances as risk factors, such as age, gender, previous overdoses, being homeless, recent prison release, and adverse life events (Rome et al., 2008). However, as there is no understanding about the relative impact of these factors on drug overdose risks, or how these factors may combine to affect the risk of suffering an overdose, the ability to predict overdoses and fatality remains poor (see (Fischer et al., 2015) for an overview).

To date, longitudinal work with substance abusers has been focused on understanding the risk factors for moving from substance use to dependence (Wittchen et al., 2008; Swendsen et al., 2009). Such work has highlighted the importance of sociodemographic and gender factors when estimating risk in this population. However, despite the considerable scope of the problem, the risk factors relating to drug overdoses have never been examined in a comprehensive, principled, and methodologically rigorous manner.

The present study proposes to address this issue by piloting a data collection form (overdose risk assessment (OdRi) questionnaire) designed to link drug overdose risk factors in a cohort of treatment-seeking opioid-dependent population in Scotland to actual incidences of fatal and non-fatal drug overdoses these individuals subsequently experience (Supplementary Material). As such, this study would help start identifying the quantitative weighting of risk factors for fatal and non-fatal drug overdoses, both in isolation and in combination. Such understanding would be fundamental to targeting specific interventions more effectively to those most at risk for suffering overdoses, with the potential to prevent such outcomes and ultimately save lives. This will also help establish algorithms to support ecologically valid user applications that can predict outcomes to risky behaviors in this population.

## Design and Methods

### Information and Ethical Governance Approvals

The OdRI study received the Caldicott Guardian approval from NHS Fife in November 2010. Following consultation with the local ethics committee and the joint Medical Research Council and National Health Service (NHS) Health Research Authority decision-making tool, the OdRi study team were notified that this study does not need ethical approval.

### Participants and Sample Size

The participants for this study are patients of the National Health Service (NHS) Fife Addiction Services, which treats approximately 1900 substance users at any one time.

In Fife, on average, there have been 30 fatal drug overdoses (drug deaths) each year over the past 6 years. Of these, around 50% were known to NHS Fife Addiction Services (Baldacchino et al., 2009; Baldacchino et al., 2010; Frisher et al., 2012; Bartoli et al., 2014). Therefore, during a data collection period of 12 months, it was anticipated that approximately 10 individuals (of the 600) would suffer a fatal drug overdose.

The anticipated numbers of non-fatal overdoses are somewhat more difficult to estimate. The Scottish Ambulance Service attend around 15 non-fatal overdoses (illicit and licit) each week in Fife with a guesstimate that only about 30% of these are individuals known to Fife NHS Addiction Services. Therefore, over a 12-month period, it was estimated that around 84 non-fatal drug overdose events were likely to occur in individuals known to Fife NHS Addiction Services (note that these are overdose incidents, not number of individuals—i.e., a single individual is likely to suffer repeated overdoses). One longitudinal study of a cohort of Scottish drug users receiving treatment for substance use disorder has found that 49% of the sample had overdosed at least once in the past, and 11% had done so in the past 3 months (McKeganey, 2008).

For the purpose of this pilot study, 640 individuals that were referred to NHS Fife Addictions Services for opioid dependence completed an OdRi questionnaire during their initial assessment between 2010 and 2012. These OdRi data were then followed up during the subsequent 5-year period for incidents of fatal and non-fatal drug overdoses and additional proxy measures of morbidity and mortality as indicated through the linkage of clinical datasets of the cohort studied.

### Overdose Risk Assessment Questionnaire

Overdose risk factors initially identified through a systematic review as “individual,” “situational,” and/or “organizational” risk factors were subcategorized into the following:

1) Personal and situational
• Emotional trauma: items 18–20;
• Physical health: items 9 and 21;
• Mental health: items 6, 7, 15, and 22;
• Extrinsic stress and heavy intoxication: items 5, 14, and 17;
• Experience of overdose recently: items 9–11;
2) Organizational;
• Lack of treatment: items 1, 2, 4, 12, 13, 16, and 24;
• Medication-assisted treatment (MAT): items 3 and 25;
• Homeless: items 8 and 23;

Additionally, as part of preparing an EMCDDA report (Robertson, 2010) on identifying and quantifying overdose risk factors, a Delphi study was also undertaken in order to cross validate the above categories from the systematic review. Based on this methodology, an overdose risk assessment (OdRi) questionnaire was designed (Humphris et al., 2013; Fischer et al., 2015).

This OdRi questionnaire is a 25-item self-reported measure assessing risk of fatal and non-fatal overdoses. Each item is rated from 0 (No) to 1 (Yes), and a higher score indicates a higher risk of overdose (Supplementary Material: OdRi questionnaire).

### Data Linkage

All treatment-seeking opioid-dependent users attending NHS Fife Addiction Services completed this overdose risk assessment (OdRi) questionnaire with a clinical staff member. These data were inputted into an NHS electronic system and then deposited, in an anonymized and coded electronic format, into the Health Informatic Centre (HIC) Safe Haven (University of Dundee, Health Informatic Centre (HIC), 2015) for it to be subsequently interrogated by the researchers of this pilot study within a time-limited period. HIC Services is a University of Dundee research support unit within the Farr Institute-Dundee, in collaboration with NHS Tayside and NHS Fife.

This database was expanded through linkage processes to include overdose events which these individuals experience over the following 5-year period. Information about overdoses was obtained from the A&E and hospital discharge records (for non-fatal overdoses) and procurator fiscal (for fatal overdoses). Other datasets used within the Health Informatic Centre (HIC) safe haven include 1) Scottish Morbidity Register (SMR) 01 and SMR04 datasets which register all hospital medical and psychiatric admissions, respectively, and 2) SMR25a/b which records new treatment episodes for substance misuse. Demographic data were also collected, including the Scottish Index of Multiple Deprivation (SIMD) (1= most deprived and 10= most affluent). The CHI (Community Health Index) number, a unique patient identifier, was used to link healthcare records to the abovementioned datasets held within the HIC.

All relevant data were anonymized for the researcher when conducting the analysis.

### Statistical Analysis

Stata 14 (Stata Corporation, College Station, TX, United States, 2015) was used for data management and statistics. The data analyzed were based on a factor analysis followed by logistic regression in order to gain initial insights into the relative strength of the individual risk factors in predicting fatal and non-fatal drug overdoses.

Before operating the explanatory factor analysis, the Kaiser–Meyer–Olkin (KMO) test and the Bartlett’s test of sphericity were used to evaluate the factorability. We opted for the exploratory factor analysis with oblique rotated (Promax) tetrachoric correlation matrix in order to collapse the questionnaire items into interpretable underlying factors. This approach was retained because of the binary format of the OdRi questionnaire items (Muthén, 1978; Muthén and Hofacker, 1988; University of Dundee, Health Informatic Centre (HIC), 2015). Only items with a communality above 0.4 (Osborne et al., 2008) and loading factor >0.4 were retained in the Results section. The three factors retained were as follows:

1. Illicit drug (usually heroin and benzodiazepine) and alcohol use and lack of treatment
2. Mental health and emotional trauma
3. Chronic drug use and frequent overdose

Factor retention was based on their interpretability along with the scree plot examination (Cattel, 1966) and Kaiser criteria of Eigenvalue >1 (Kaiser, 1960). The reliability of items was examined by computing the Cronbach’s alpha coefficient (Santos, 1999).

For fatal drug overdose, all 25 items were included in the exploratory factor analysis, while for non-fatal drug overdose events, the same explanatory factor analysis was repeated with the exclusion of items 9 to 11. Logistic regression was used to assess factors predicting fatal and non-fatal overdoses. In adjusted analysis models, age and sex were introduced as covariates. Risk was expressed as odds ratio (OR) with 95% confidence interval [95% CI]. Alpha risk was set at 5%.

## Results

### Demographics

Completed data from 640 participants were used for the current analysis. The average age of participants was 42.2 ± 0.3 years, and 30.2% of them were women. The mean Scottish index of multiple deprivation (SIMD) was 2.9. Of the participants, 8.6% (*n* = 55) died due to an fatal drug overdose (drug death), 38.2% experienced at least one non-fatal drug overdose across their life span, and 6.9% experienced a non-fatal drug overdose during the last year, while 2.2% experienced two or more non-fatal drug overdoses during the last year. All steps that were undertaken to develop and validate the questionnaire were reported as a Supplementary Material (Supplementary Figure S1).

### Fatal Drug Overdose

EFA suggested a three-factor solution accounting together for 75% of the total variance.

*Internal reliability*: Overall, the questionnaire showed a questionable reliability level of 0.645. Subgroup analysis of Factors 1–3 (Table 1) showed a satisfactory level for the item belonging to the first factor (illicit drug use), while reliability was questionable too low for the second (mental health and emotional trauma) and the third (chronic drug use and overdose) factors.

**TABLE 1:** Exploratory factor analysis and internal consistency for fatal drug overdose episodes.

		Fatal drug overdose			
OdRi questions	Items	Factor 1 (illicit drug use and lack of treatment)	Factor 2 (mental health and emotional trauma)	Factor 3 (chronic drug use and frequent overdose)	Cronbach’s α coefficient
1	Current heroin user (smoke and snort)	0.71			0.70
2	Current intravenous drug use	0.71			
3	Current prescription for opiate dependence (methadone, buprenorphine, and suboxone)	−0.69			
13	Having to use increasing amounts of drugs to become intoxicated	0.59			
16	Tends to use alone	0.83			
25	At the beginning of treatment	0.64			
7	Poly use of CNS depressants (include prescription psychotropic medication, that is, antidepressants and antipsychotics)		0.63		0.60
18	Domestic abuse past or present		0.73		
19	Domestic abuse past or present		0.75		
20	Past termination or miscarriage (women only)		0.56		
22	Mental health diagnosis		0.62		
10	Has been in prison, hospital, or residential detox in preceding month, or currently on detox prescription			0.63	0.34
11	Has overdosed accidentally/intentionally two or more times in the past year			0.72	
12	Has been using drugs for more than 5 years			−0.69	

*Predictability of fatal drug overdose*: Results displayed in Table 2 showed that the increase of the Factor 2 (Mental health and emotional trauma) score by one unit increases the risk of fatal drug overdose by nearly two-fold.

**TABLE 2:** Logistic regression for the association between latent factors with fatal and non-fatal drug overdoses.

	Crude analysis		Adjusted analysis	
	OR	95% CI	OR	95% CI
	Fatal drug overdose			
Factor 1(illicit drug use and lack of treatment)	0.71	0.29–1.74	0.70	0.28–1.72
Factor 2 (mental health and emotional trauma)	1.76	0.98–3.17	1.94	1.03–3.63
Factor 3 (chronic drug use and frequent overdose)	0.61	0.21–1.77	0.61	0.21–1.79
	Non-fatal drug overdose (ever)			
Factor 1 (illicit drug use and lack of treatment)	1.85	1.13–3.04	1.80	1.09–2.97
Factor 2 (emotional trauma)	1.99	1.23–3.20	2.74	1.51–4.95
Factor 3 (chronic drug use and mental health)	3.18	1.97–5.15	3.39	2.07–5.57
	Non-fatal drug overdose (one in a past year)			
Factor 1 (illicit drug use and lack of treatment)	3.73	1.60–8.69	3.82	1.61–9.06
Factor 2 (emotional trauma)	2.09	0.91–4.81	2.23	0.78–6.34
Factor 3 (chronic drug use and mental health)	3.53	1.58–7.87	3.13	1.36–7.19
	Non-fatal drug overdose (one or more in a past year)			
Factor 1 (illicit drug use and lack of treatment)	8.69	2.12–35.5	7.54	1.81–31.4
Factor 2 (emotional trauma)	2.89	0.73–11.4	2.74	0.48–15.72
Factor 3 (chronic drug use and mental health)	0.64	0.11–3.64	0.56	0.09–3.45

### Non-Fatal Drug Overdose

EFA suggested a three-factor solution accounting together for 97% of the total variance.

*Internal reliability*: The first factor showed an acceptable reliability (*α* = 0.70), while the second (*α* = 0.65) and third (*α* = 0.59) factors showed questionable and poor reliability, respectively (Table 3).

**TABLE 3:** Exploratory factor analysis and internal consistency for non-fatal drug overdose episodes.

Non-fatal drug overdose					
		Factor 1 (illicit drug use and lack of treatment)	Factor 2 (emotional trauma)	Factor 3 (chronic drug use and mental health)	Cronbach’s α coefficient
1	Current heroin user (smoke and snort)	0.69			0.70
2	Current intravenous drug use	0.73			
3	Current prescription for opiate dependence (methadone, buprenorphine, and suboxone)	−0.78			
13	Having to use increasing amounts of drugs to become intoxicated	0.61			
16	Tends to use alone	0.70			
25	At the beginning of treatment (titration prescription)	0.68			
18	Domestic abuse past or present		0.82		0.65
19	Emotional/sexual abuse past or present		0.70		
20	Past termination or miscarriage (women only)		0.78		
7	Poly use of CNS depressants (include prescription psychotropic medication, i.e., antidepressants and antipsychotics)			0.78	0.59
22	Mental health diagnosis			0.74	

*Predictability of non-fatal drug overdose*: According to Table 2, the regression analysis showed that all the three factors are significantly associated with non-fatal drug overdose (ever), while only the first and the third factors are significantly associated with experiencing a drug overdose during the past year. The increase of the Factor 1 (illicit drug use) score by one unit increases the risk of more than one overdose during the past year by three-fold.

## Discussion

### Summary and Questionnaire Validity

In this study, data from 640 patients were collected from the National Health Service (NHS) Fife Addiction Services using the OdRi questionnaire. This pilot study aimed to start identifying the quantitative weighting of risk factors for fatal and non-fatal drug overdoses.

The exploratory factor analysis, tetrachoric correlation matrix, for fatal overdose identified three factors, namely, Factor 1 “*illicit drug use and lack of treatment*,” Factor 2 “*mental health and emotional trauma*,” and Factor 3 “*chronic drug use and frequent overdose*.” A similar number of factors were identified for non-fatal overdose, but the mental health item was loaded on a third factor along with drug use–related items. The overall questionnaire’s (all items) internal consistency was questionable; however, after running factor analysis, we found that items of the Factor 1 (in both fatal and non-fatal overdose data analysis) items reached an acceptable value. Items of Factors 2 and 3 fell below the requirement for internal consistency, which could be attributed to the low number of items or due to the poor interrelatedness between items (Tavakol and Dennick, 2011). It is unexpected that the obtained internal consistency of both Factors 2 and 3 could be attributed to constructs’ heterogeneity. Indeed, a difference in participants’ characteristics may evolve a large interindividual variability and then impact the homogeneity of measurement items (Tavakol and Dennick, 2011). However, in our study, we have very few measurements of individual characteristics. For example, the subjects’ education level was not measured. Of note, the questionnaire’s multidimensionality might contribute to the poor internal consistency of certain items (Tavakol and Dennick, 2011). Beyond that, the internal consistency is proportional to the number of items, and the low item number might alter the questionnaire performances. Bernardes Santos et al. (Santos et al., 2009) indicated that the combination of scales assessing independent constructs might introduce bias in internal consistency interpretation. 

### Interpretation

This study showed that mental health factors were positive predictors of both fatal and non-fatal overdoses. In the available literature, individuals suffering from mental health have been reported to be more likely to experience drug abuse and then to have an increased risk of opioid overdose (Cicero and Ellis, 2017). Specifically, depression was associated with fatal (Foley and Schwab-Reese, 2019) and non-fatal (Tobin and Latkin, 2003) overdoses. Noticeably, our results were in agreement with a growing body of literature showing that early life stress is associated with both forms of overdoses (Braitstein et al., 2003; Cutajar et al., 2010; Khoury et al., 2010; Lake et al., 2015). For example, participants from two Canadian cohort studies (*n* = 1,679) found that physical, sexual, and emotional abuse during childhood increased (1.5-fold) the risk of non-fatal overdose (Lake et al., 2015). These findings highlight the need for systematically screening for mental health and emotional trauma in order to predict fatal and non-fatal overdoses. While limited importance has been given to the mental health component in drug overdose developed questionnaires at the time of study, Fendrich et al. (2019) suggested integration of validated questionnaires for mental health rather than introducing few self-reported items as in the study by Butler et al. (2008). Indeed, Fendrich et al. (2019) have combined four validated scales, for depression (PHQ-9 questionnaire), severe anxiety (Beck Anxiety Inventory), post-traumatic stress disorder (Mini-International Neuropsychiatric Interview), and psychosis (Behavior and Symptom Identification Scale-24). They found that individuals with severe depression, post-traumatic stress disorder, or psychosis have an increased risk (2.5-fold) to experience a drug overdose during the previous 3 months.

In comparison to Factor 2, Factor 1, that is, “Illicit drug use and lack of treatment,” was found to be a predictor of recent and lifetime non-fatal drug overdose. Individuals who are not and/or have just been stabilized in a treatment program continue to experience drug overdose. Additionally, individuals who are integrated within a drug treatment program are also at risk of further non-fatal overdose due to increasing susceptibility for overdose through reduction of individual tolerance (Pollini et al., 2006). Moreover, multi-substance use may complicate treatment and management of addiction.

Finally, it is worth to mention that there was not a significant association between age and gender with fatal and non-fatal overdoses.

### Strengths and Limitations

The study accounts on the OdRi questionnaire that drives from an exhaustive literature review for risk factors of overdose. Indeed, the questionnaire gathers several factors related to overdose, including “individual,” “situational,” and/or “organizational” ones. Second, the important number of patients enrolled in this study would increase the generalizability of the results obtained from this study. Finally, stringent criteria were used for the exploratory factor analysis and factor identification.

Our study has some limitations. The patients were not randomly selected, so no inference could be made to general population of illicit drug and substance users in Scotland. Second, the self-reported data may introduce a recall bias. Third, no validated scales were used for the assessment of specific aspects of mental health (i.e., depression and anxiety). Fourth, emotional trauma (including all forms) might be underreported. Fifth, our study includes few potential confounders (i.e., age and sex); then an extension to others such as socioeconomic level and family context should be warranted. The analyses were conducted among patients from low-income areas as mirrored by the mean Scottish index of the multiple deprivation index. Then, the strengths of association between the constructs and overdose occurrence (both fatal and non-fatal) might be different in high-income areas. Finally, the response collected about health problems was subjective as no clinical diagnosis was realized. The establishment of these data for this study could have been enhanced by using tertiary data such as clinical notes and electronic portal systems.

### Clinical and Public Health Relevance

The ultimate importance of this work lies in the potential to greatly enhance our current knowledge of the risk factors underlying drug overdoses. This is of utmost importance knowing that in Scotland, 1,339 drug-related death cases were identified in 2020 (National records of Scotl, 2021), and nowadays, it is estimated to be the highest rate in Europe. Such information would help identify individuals most at risk, facilitating more targeted and timely interventions, and thereby save lives. The understanding of the relative importance of the risk factors for suffering fatal and non-fatal drug overdoses that would be gained by the present study is also fundamental to the development of an overdose risk assessment tool. This is one of the future directions of this line of research, should the study be successful in securing funding in the future. The data collection process would be continued in Fife in order to expand the sample size to obtain more reliable results. If successful, this process could be set up in other services and regions, expanding the sample size and potential knowledge gain even further. Knowledge transfer and exchange to policy-makers, professionals, substance misuse treatment service users, the general public, families, and careers are an essential outcome of the proposed study, and the study team are very well placed to disseminate the study findings in their respective roles.

It will also be a unique opportunity to established highly predicable algorithms which can be used to establish user applications that can be therapeutic in nature and empowering for the service user. It will help build on the work initiated by the EU-funded ORION project (http://orion-euproject.com/) which established a PC-based eHealth tool. This can be further developed using a mobile digital application platform.

## Conclusion

Our study represents the first application of the OdRi questionnaire for the assessment of the overdose risk factors. Further studies are needed to assess the questionnaire’s reproducibility (test–retest approach) for internal consistency. However, our study showed that mental health and life stress conditions increase the risk of fatal and non-fatal overdoses among adult drug using treatment-seeking cohort users. Systematic screening of mental health and life stresses (including early life stress) should be encouraged to provide the necessary assistance for patients and organize a service that will be trauma-informed. Further studies should be conducted to assess the different forms of mental health problems and their association with overdose. Along with the mental health management, any intervention should promote other microlevel factors such as healthy lifestyle (i.e., healthy diet and regular physical activity). Because of the health and economic burden of drug misuse, acting at the macrolevel is necessary; indeed, that more attention should be given to substance use through an effective community-based prevention.

## Data Availability

The data analyzed in this study are subject to the following licenses/restrictions; these data are the property of the University of Dundee (Health Informatics Centre); requests to access these datasets should be directed to https://www.dundee.ac.uk/hic/hicsafehaven/.
