# Supplementary material for: Understanding Fatal and Non-Fatal Drug Overdose Risk Factors: Overdose Risk Questionnaire Pilot Study—Validation
Source: Front Pharmacol. 2021 Sep 28;12:693673. doi: 10.3389/fphar.2021.693673 (PMC8506126; doi:10.3389/fphar.2021.693673)
Supplement: Supplementary file 1 [file DataSheet1.docx]

Supplementary Material

OVERDOSE RISK ASSESSMENT QUESTIONNAIRE

Instructions:

1. Complete the pro-forma with your patient;

2. If the answer to a question is (yes) circle the score; if answer is (no) record no
score for that question;

3. Make any notes related to the answer as required e.g. mode of use, high risk
injecting, number of units of alcohol, prescription or illicit use etc
4. Add up the total score to establish the level of risk;

5. Complete management of risk plan in patient notes.

| **Item number** | **RISK FACTOR** | **Score**  **(1=Yes/0=No)** | **ADDITIONAL INFORMATION** |
| --- | --- | --- | --- |
| 1 | Current heroin user (smoke, snort) |  |  |
| 2 | Current intravenous drug use |  |  |
| 3 | Current Prescription for opiate dependence (methadone, buprenorphine, suboxone) |  |  |
| 4 | Is **not** on supervised consumption of prescribed substitute medication. |  |  |
| 5 | Also drinks alcohol (please detail amounts) |  |  |
| 6 | Current use of benzodiazepines (mode of use, amounts whether prescribed or illicit) |  |  |
| 7 | Poly use of CNS Depressants (include prescription psychotropic medication, i.e antidepressants, anti psychotics) |  |  |
| 8 | Has been in prison, hospital or residential detox in preceding month, or currently on detox prescription |  |  |
| 9 | Has overdosed accidentally/intentionally (ever) |  |  |
| 10 | Has overdosed accidentally/intentionally once in past year |  |  |
| 11 | Has overdosed accidentally/intentionally two or more times in past year |  |  |
| 12 | Has been using drugs for more than 5 years |  |  |
| 13 | Having to use increasing amounts of drugs to become intoxicated |  |  |
| 14 | Enjoys or wants to feel heavily intoxicated |  |  |
| 15 | Currently experiencing severe low mood, depression or suicidal thoughts |  |  |
| 16 | Tends to use alone |  |  |
| 17 | Recent Bereavement ( in past year) |  |  |
| 18 | Domestic Abuse past or present |  |  |
| 19 | Emotional/sexual abuse past or present |  |  |
| 20 | Past termination or miscarriage (women only) |  |  |
| 21 | Has physical health problems (e.g hepatitis, cirrhosis, respiratory problems) |  |  |
| 22 | Mental health diagnosis |  |  |
| 23 | Homeless/roofless |  |  |
| 24 | Sporadic attendance at appointments |  |  |
| 25 | At beginning of treatment (titration prescription) |  |  |
| **Total score** | |  |  |


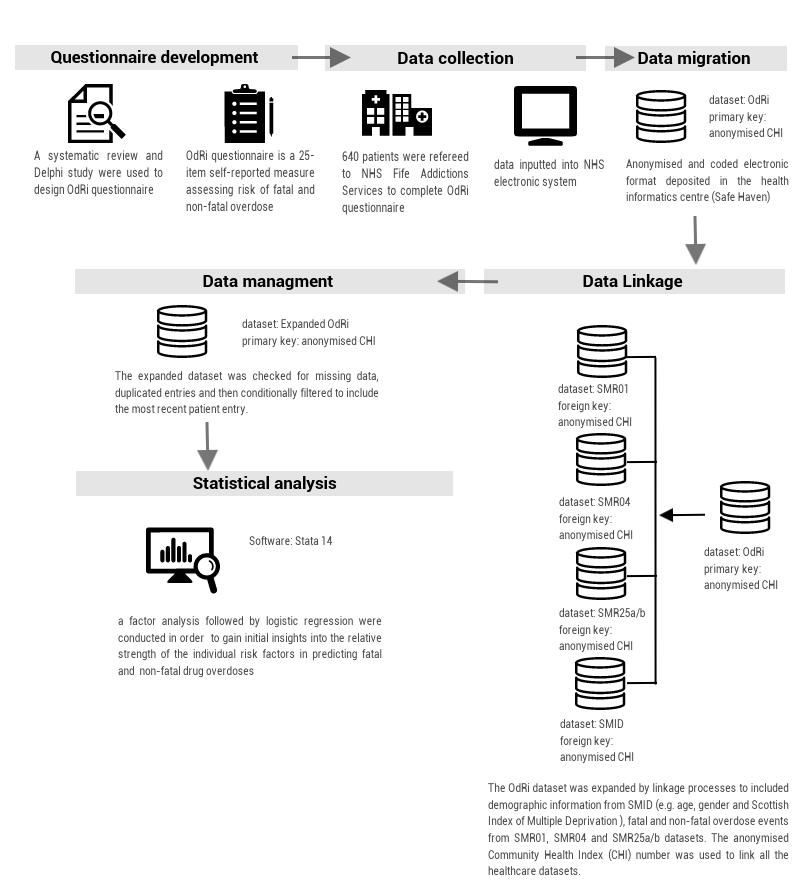


**Figure S1**. flow diagram for all the steps that were undertaken to develop and validate the questionnaire.
